# Intramuscular Insertion of a Radiofrequency Microneedling Device for Facial Rejuvenation: A New Technique and Case Reports

**DOI:** 10.1093/asjof/ojz035

**Published:** 2020-02-11

**Authors:** Andrew N Kornstein

## Abstract

Skin laxity and dynamic rhytids that signify facial aging are due, in part, to atrophic changes and volume redistribution in the underlying layers of the face: the bone and soft tissue. A microneedling device that delivers bipolar radiofrequency (RF) energy directly to the reticular dermis has been shown to yield rejuvenated, tighter skin in 100% of patients. Herein, the author describes a modification to the standard dermal technique in which the skin is gently gathered with the nondominant hand, and the microneedling device is tilted to deliver RF energy to the mimetic muscles—including the frontalis, orbicularis oculi, and orbicularis oris—as well as the dermis. Muscle penetration was inferred by intraoperative bleeding and postoperative bruising, neither of which is typical of standard RF microneedling. Preliminary findings suggest that the modified procedure may yield greater aesthetic benefits than achieved with dermal application alone, including subtle lifting of the brow and oral commissure, upper-lip shortening with vermilion eversion, tightening of the lower-lid fat pads, and reduction in lateral-canthal rhytids. Although this modified technique will need to be optimized and evaluated in large, controlled studies, the initial results presented herein are encouraging.

**Level of Evidence: 5:**

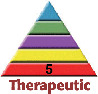

Aging is a composite of several processes: reductions in hydration, collagen content, and elasticity of the skin; loss and redistribution of soft-tissue volume; and bone resorption and remodeling.^1^ These changes are influenced, at least in part, by altered hormonal levels and the damaging effects of chronic sun exposure and smoking.^1^ Based on results of my and others’ work, I believe that a reduction in circulation is responsible for age-related atrophy of the soft and hard tissues and that this phenomenon explains the antiaging effects of grafted adipose cells.^2,3^ These atrophic changes result in depletion of underlying support and subcutaneous fullness, exposing the intrinsic tone of the facial muscles and ultimately yielding skin laxity and deep rhytids.^1,4^ Many patients present for aesthetic treatment to address sagging facial features, which appear as perpetual expressions of sternness, fatigue, or sadness.^5^ Despite a plethora of treatments for facial rhytids and laxity—including surgical reconstruction, dermabrasion, chemical peels, laser resurfacing, neuromodulators, and injectable fillers—demand continues for modalities that maximize aesthetic improvement while minimizing risk, downtime, and need for repeat sessions.

## Profound Radiofrequency Microneedling

Nonablative radiofrequency (RF) technology utilizes an electric current to transfer focal thermal energy. The Profound bipolar RF system (Candela Medical, Wayland, MA) enables the surgeon to deliver fractional thermal injury directly to the reticular dermis by means of 5 pairs of electrode-fitted, 32-gauge microneedles, spaced 1.25 mm apart. The device senses temperature and electrical resistance in real time and modulates power as needed to maintain a preselected intralesion temperature over a range of tissue impedances (eg, 200–3000 Ω).^6–9^ To avoid damage to the skin surface, the system maintains an epidermal temperature of 10°C by means of a Peltier module.^7^

The Profound RF device applies a 3- to 4-s time-at-temperature pulse at 67°C, which causes collagen molecules in the dermis to partially denature and triggers an anabolic wound-healing response in which fibroblasts increase the concentration of collagen, elastin, and hyaluronic acid in the skin.^6–8^ The expression profile of matrix metalloproteinases in the 4 weeks following intradermal treatment supports the hypothesis that fractional RF promotes a unique tissue response in which processes involved in dermal remodeling are sustained, with little degradation of preexisting collagen and elastin.^8^

## Clinical Results

In clinical studies, significant aesthetic benefits have been obtained with bipolar RF microneedling. In 2013, Alexiades-Armenakas et al^6^ carried out a prospective study of 100 patients with mild-to-severe facial and neck rhytids and laxity. Intradermal lesion temperatures of 62°C to 78°C were applied for 3 to 5 s, and patient photographs were graded by blinded evaluators. At 6 months postoperatively, the investigators found mean improvement of 25.6% on the Fitzpatrick Wrinkle Scale and 24.1% on the Alexiades Laxity Scale.^6^ The study population had response rates of 100% for rhytids and 95% for laxity overall, and in subgroup analysis, rhytid and laxity response rates were 100% for patients treated at 66.7°C for 4.2 s.^6^ In a subsequent 3-arm study,^10^ the authors demonstrated a 100% response rate for rhytids and laxity with treatment parameters of 67°C and 3 s. Intradermal treatment of 15 patients produced 37% of the mean laxity improvement of facelift, as graded by 5 blinded evaluators.^11^ Other investigators demonstrated that bipolar RF microneedling improved the elastometry characteristics of the jawline and cheek in 44 patients, yielding skin that appeared 2.6 years younger, on average, as graded by physicians.^12^

## Mimetic Musculature

Few options are available for managing the aging facial musculature, and age-related changes in this layer of the face are not well characterized. Treatment of deep dynamic rhytids often involves selectively weakening the underlying mimetic muscles with a neuromodulator (eg, Botox Cosmetic, Allergan).^13^ However, neuromodulator treatment is associated with swelling, bruising, and headache in approximately a quarter of patients and occasionally produces undesirable muscle weakening and ptosis.^13^ Multiple sessions at approximately 6-month intervals are needed to sustain the effects of neuromodulator treatment, and an immune response in some patients hinders or precludes the aesthetic benefits of subsequent applications.^13^

## A Modification to RF Microneedling

After years of applying the Profound RF device dermally according to the manufacturer’s recommendations, I reasoned that an additional benefit might be achieved if both the reticular dermis and the mimetic muscles were subjected to microneedle penetration and fractional RF energy. In the lower eyelid and “crow’s feet” as well as the upper lip and commissure, the musculature and skin are intimately associated and move as a unit, making intramuscular delivery of RF energy relatively simple. I considered that an anabolic repair process might be triggered in the superficial muscles and that desirable lifting and tightening could potentially be achieved in 2 layers of the aging face in a single, minimally invasive procedure.

## Patients and Surgical Procedures

Patients who underwent intramuscular treatment with bipolar RF microneedling presented with mild to severe rhytids and laxity of the face and neck. Those with a history of superficial facial implantation, smoking, bleeding disorders, or a compromised immune system were considered less suitable for this procedure, and patients with scarring diathesis were excluded.

All patients depicted in this study provided written informed consent. Patients were photographed at rest with a Nikon D3100 camera system (Nikon, Tokyo, Japan) equipped with a Nikon 100-mm macro lens, a Nikon Speedlight SB-700 flash, and a Gary Fong flash diffuser. Photographs were taken before the procedure and during follow-up, with the same camera, flash system, and background; however, the amount of ambient light was not constant.

RF microneedling was carried out in a single session. Patients received Pro-Nox nitrous oxide sedation (Carestream Medical, Altamonte Springs, FL), and tumescent local anesthesia was achieved with a dilute solution comprising 30 mL of 1% lidocaine with epinephrine (1:200,000), 20 mL of 1% plain lidocaine, 10 mL of bicarbonate, and 50 mL of normal saline. Infiltration with local anesthesia is known to lower tissue impedance in some conditions; ^7^ however, the Profound RF feedback algorithm actively monitors impedance and corrects pulses accordingly to maintain the preselected intralesion temperature. In my experience, the added tumescent volume results in less patient discomfort and decreased bruising.

The Profound RF device was fitted with the dermal cartridge and was set to deliver 3.8-s pulses to a tissue temperature of 67°C. Dermal treatment then was carried out from the frontal hairline to the clavicle, the sternocleidomastoid on one side to the sternocleidomastoid on the other side, bilaterally from one preauricular region to the other, and from the temporal crest on one side to the temporal crest on the other side. The only area not treated was the temporal region from the zygomatic arch to the temporal crest.

A single-pass treatment was performed in all mentioned areas except over the frontalis, orbicularis oculi, orbicularis oris, and jowl. There, a double-pass, intramuscular procedure was conducted in a crosshatch pattern. Specifically, RF treatment of the upper lip was carried out laterally to medially and then superiorly to inferiorly (ie, toward the vermilion, up to, and including the mucocutaneous junction). For the lower lid and crow’s feet, the procedure ran laterally to medially and then inferiorly to superiorly (toward the orbital-rim margin). In the upper lip and commissure, treatment was focused in the mucocutaneous junction. Tightening of the tubular muscle underlying this area encourages lip eversion. RF delivery also was performed meticulously around the oral commissure; this maneuver elevated the corners of the mouth while tightening the buccal region owing to the anatomic attachment of the buccinator muscle to the oral musculature.

For intradermal treatment, the cartridge was placed flush with the skin, with countertraction applied by the surgeon’s nondominant hand to create a flat surface.^7^ The device then deployed the microneedles at an angle of 25°; the electrode tips penetrated to an approximate depth of 1 to 2 mm.^6^ Each 6-mm-long electrode is insulated along the proximal 3 mm, leaving only the distal 3 mm exposed. To avoid blistering or punctate atrophy, RF energy was not applied until it was determined that the needles were correctly inserted in the skin.^6^

**Figure 1.**
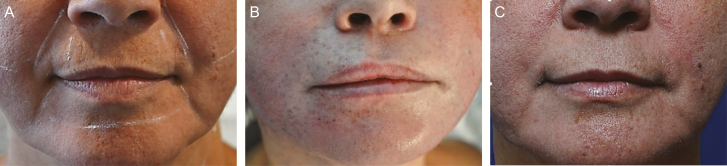
(A) This 52-year-old woman presented with facial laxity and received Profound radiofrequency (RF) rejuvenation of the entire facial skin, including the forehead, neck, and the periocular, perioral, and mandibular regions. She also underwent intramuscular RF treatment of the orbicularis oris, which involved 75 insertions into the upper lip and 65 insertions into the lower lip. (B) The patient is depicted intraoperatively, after infiltration of local anesthesia and treatment of the upper and lower lips on the right side of the face. Note the lifted skin and upper-lip eversion on the right side, compared with the untreated left side. Local anesthetic was injected bilaterally, so the only difference is the energy delivered unilaterally to the orbicularis oris. (C) Five months postoperatively, there is aesthetic improvement in the perioral region, including eversion of the lateral oral mucosa of the upper and lower lip, shortening of the lateral upper lip, enhanced definition of the white roll of the upper and lower lip (including the cupid’s bow), and fewer vermilion rhytids (where there is no dermis to rejuvenate).

For intramuscular treatment, the skin was gently pinched with the surgeon’s nondominant thumb and forefinger, lifting the muscle from the subjacent bone or intraoral mucosa. The heel of the cartridge was elevated at an approximately 30° incident angle to the pinched skin to allow for deeper access of the microneedle array. Penetration of the muscle was inferred by intraoperative bleeding (Figure 1), which is minimal or absent when RF microneedling is limited to the dermis. Extreme care was exercised when treating areas of thinner skin, such as the lateral part of the lower lip, the lower lid, and the crow’s feet. Deep insertion of the electrodes into these areas can result in intraoral puncture or contact with the bone of the orbital rim.

Profound RF was utilized to treat the frontalis in patients with thinner forehead skin only. The frontalis in patients with thicker skin and the platysma in all patients are not amenable to this technique because the target muscles are too deep. In the future, it may be possible for the manufacturer to overcome this limitation by producing an intramuscular cartridge with slightly longer microneedles. As atrophy of the frontal bone and fat pad occurs with aging, there is a compensatory redundancy of the frontalis that develops because this muscle must be in a hyperdynamic state to achieve proper brow position and maintain upper-lid (levator) function. The frontalis was stimulated by RF microneedling with the aim of reducing redundancy and diminishing these hyperdynamic rhytids. Patients were treated in transverse and vertical rows bilaterally from one temporal crest to the other and from the anterior hairline to the caudal eyebrow.

Other investigators have suggested that the distance between adjacent electrode deployments can be adjusted based on severity of wrinkles and skin ptosis, with 4- to 8-mm distances utilized for mild-to-moderate rhytids/laxity and 3 to 4 mm for severe rhytids/laxity.^6^ I inserted the microneedle array intradermally at intervals of approximately 4 mm (2 to 3 insertions per centimeter). For intramuscular treatment, I applied a denser insertion pattern. Patients received approximately 100 dermal insertions of the Profound RF device on each side of the lower face and 100 to 150 in the neck’ as well as an average of 79.5 intramuscular insertions in the forehead, 33.9 per lower lid, and 26 in the upper lip.

Videos 1–5 demonstrate Profound RF intramuscular treatment of the forehead, lower lid, oral commissure, lower lip, and upper lip, respectively. A detailed description of each video is described in Supplementary Material.

## Postoperative Care

Patients were given a 7-day course of herbal supplements, including 30 sublingual arnica tablets and 42 enteric-coated bromelain complex tablets (Scarguard Labs, LLC; Great Neck, NY). Patients were advised to apply Aquaphor ointment (Eucerin, Wilton, CT) 3 times during the first 24 h after treatment and to wash with mild soap and apply Aquaphor 1 additional time at 24 h posttreatment. After this, patients were instructed to apply arnica gel (eg, Boiron, Newtown Square, PA). Patients also were encouraged to undergo laser treatment 72 h after intramuscular RF microneedling to reduce bruising, which can be substantial with treatment of the facial muscle.

## Assessment of Satisfaction

Patient satisfaction and success of the procedure were assessed informally by the following:

1. Showing the patient her preoperative and postoperative photographs side by side and asking her to indicate her level of subjective satisfaction with the outcome.
2. Qualitative findings that patients treated intramuscularly with Profound RF often have a reduced requirement for neurotoxin (except in the glabella) and no need for a surgical lip lift or lip filler.

## Special Considerations

The intramuscular modification involves bunching, or pillowing, the tissues with the nontreating hand to prevent needle damage due to bony impact. Pillowing the tissue also helps avoid piercing the oral mucosa when treating the orbicularis oris (although, at 68°C, infection is not a concern). The globe is protected by placing the nontreating index finger along the superior (brow) and inferior (lower lid) orbital rim; this prevents both penetration into the orbit and damage to the pretarsal section of the orbicularis oculi.

Patients who receive frontalis treatment occasionally experience transient anesthesia of the supratrochlear and supraorbital nerves that persists for several weeks. No patient has experienced any other untoward event, such as frontal branch injury upon treatment of the lateral canthus or brow. In general, patients who undergo the modified intramuscular technique have more bruising and downtime than do patients who receive only dermal treatment.

## CASE REPORTS

### Case 1

**Figure 2.**
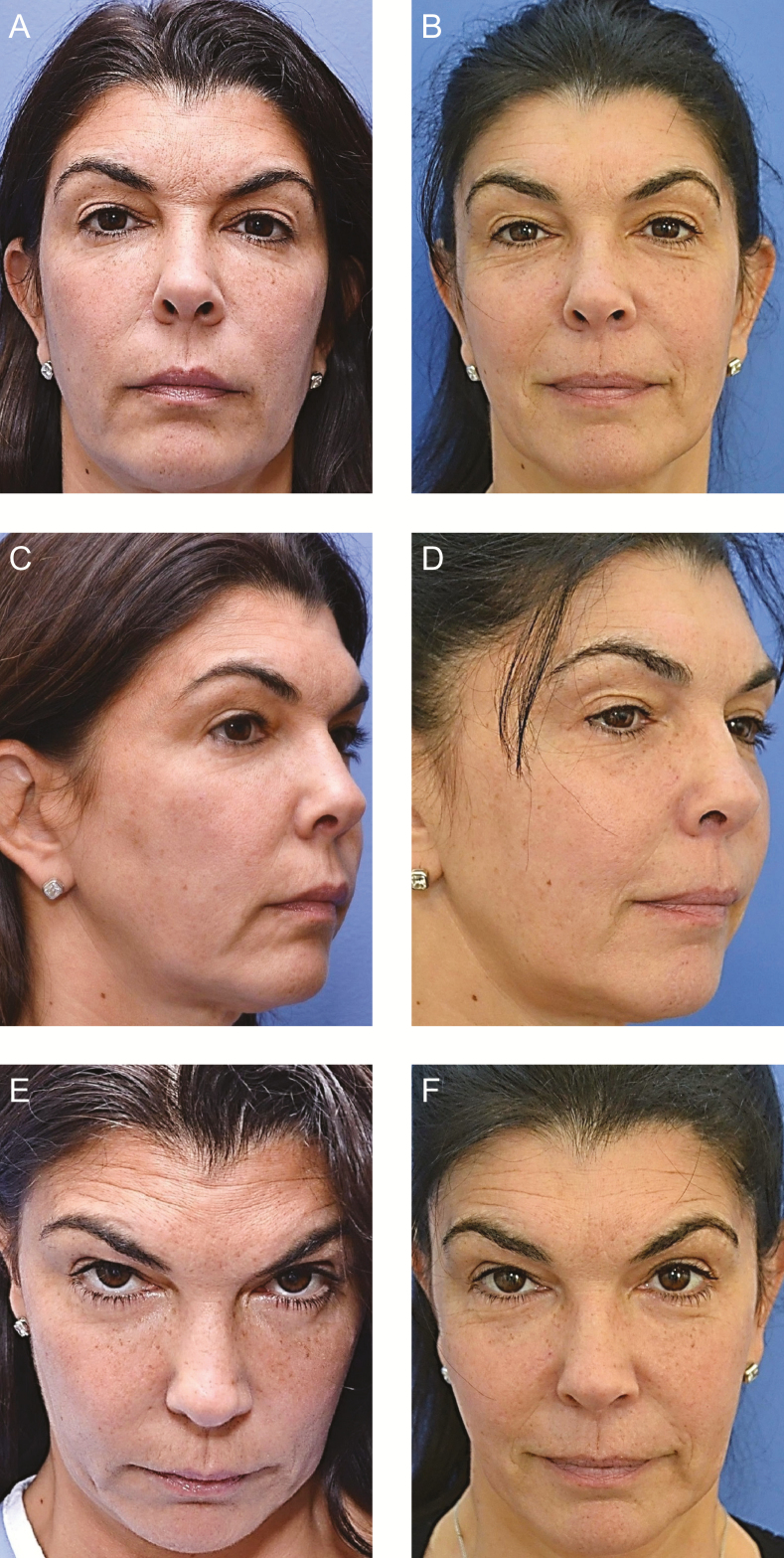
(A, C, E) This 53-year-old woman (Case 1) underwent intramuscular Profound RF treatment of the frontalis (54 insertions) and orbicularis oris (upper lip, 53 insertions; lower lip, 29 insertions). (B, D, F) Nine months after the procedure, the patient’s forehead rhytids were reduced and the medial brow was elevated, lifting the skin excess of the medial upper eyelid. The position of the oral commissure was lifted, and there was increased vermilion show, especially of the lateral upper lip. Although the patient initially appears to be smiling in the postoperative photographs, all views portray a consistent position of the oral commissure (ie, non–muscle-activated smiling). That is, the perceived change in facial expression is due to the tightening effect of the RF microneedling treatment.

This 53-year-old woman presented in March 2018 with moderate skin laxity (Figure 2). The patient had minimal discomfort during treatment with the Profound RF device and experienced edema and bruising that resolved spontaneously by 10 days postoperatively. The patient indicated that she was satisfied with the postoperative results, and she resumed her routine activities by 3 days postoperatively.

Nine months postoperatively, the patient’s brow laxity was improved, her transverse forehead rhytids were ameliorated, and her upper lip was elevated and everted with vermilion show extending to the commissure (Figure 2). In general, her face appeared youthful with a pleasant expression at rest.

### Case 2

**Figure 3.**
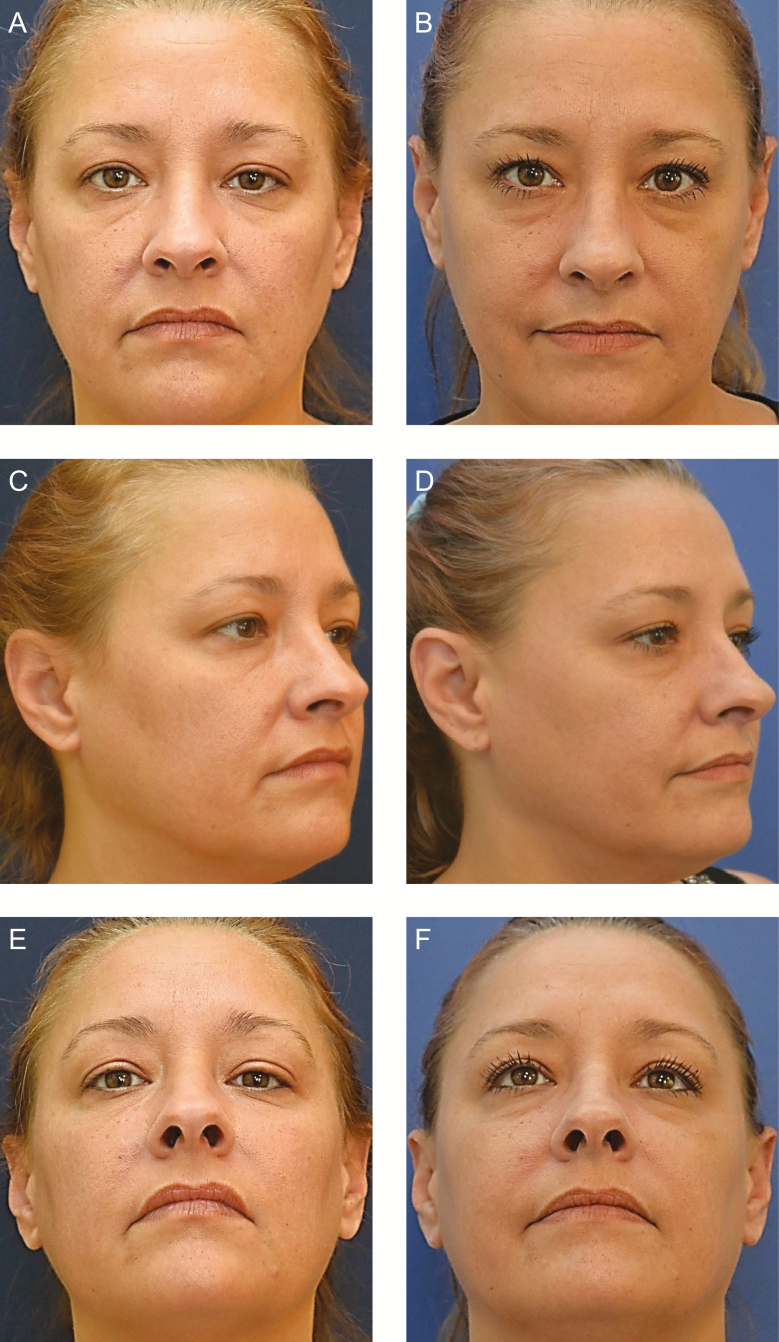
(A, C, E) This 47-year-old woman (Case 2) underwent Profound RF rejuvenation of the forehead, lower lids, upper and lower lips, mandibular border, and neck (total insertions, 384). (B, D, F) One month postoperatively, the transverse and glabellar rhytids of the forehead were less pronounced, and the animation-induced rhytids of the lower lid were softened. The position of the oral commissure was lifted, and the facial expression was neutral, rather than sad or frowning.

This 47-year-old woman presented in July 2018 with moderate laxity of the face and neck (Figure 3). The patient had orbicularis oculi redundancy and mild festoons. The eyelid area comprises 3 concentric circular components: a pretarsal involuntary blink, a preseptal involuntary blink with voluntary closure, and an orbital voluntary contraction. Care was exercised to proceed with Profound RF treatment only to the orbital rim, avoiding the pretarsal and preseptal components.

The patient experienced only mild discomfort during RF microneedling. She was able to return to her normal activities by 3 days postoperatively. During the healing process, the patient had transient edema and bruising.

At the 1-month follow-up visit, the patient had less prominent under-eye laxity and more everted lips with vermilion show laterally (Figure 3). Her skin appeared hydrated and toned. She expressed a high level of satisfaction with the cosmetic outcome. Video 6 depicts this patient making similar facial expressions before treatment and 1 year after treatment. The posttreatment effects shown in the video are described in Supplementary Material.

## DISCUSSION

**Figure 4.**
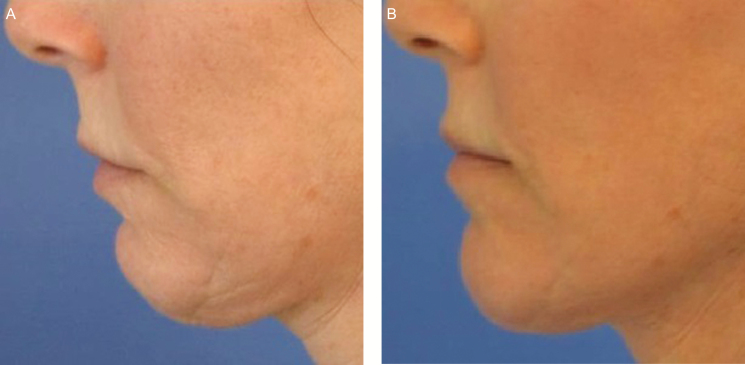
(A) This 60-year-old woman underwent Profound RF dermal rejuvenation of the forehead; periocular, perioral, and mandibular regions; and neck. In addition, she received RF microneedling placed intramuscularly, with 60 insertions into the upper lip and 50 into the lower lip. (B) Six months after the procedure, the patient had upper-lip shortening and eversion, with improved vermilion show. The reduced upper-lip length also resulted in activation of the mentalis to elevate the lower lip, ensuring oral competence. The patient stated that she is happy with her lip volume and with the harmonization of her upper and lower lips. The addition of filler is possible but no longer required.

**Figure 5.**
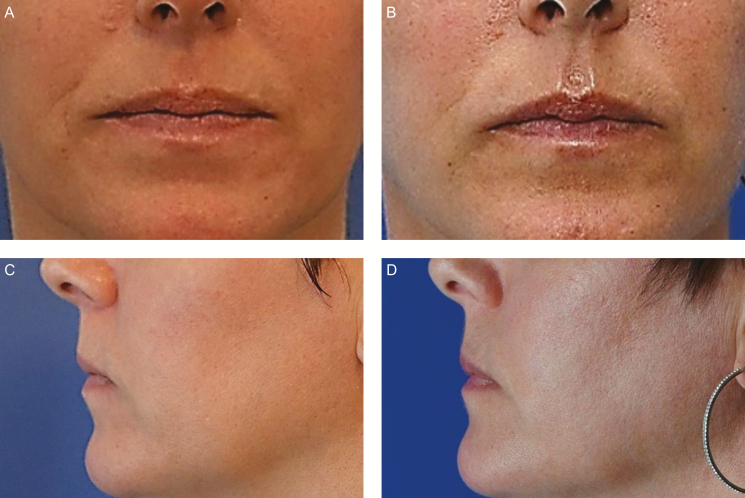
This 47-year-old woman presented with facial rhytids and laxity and underwent Profound RF treatment for rejuvenation of the forehead; periocular, perioral, and mandibular regions; and neck. She received 30 intramuscular insertions in the upper lip and 45 in the lower lip (ie, orbicularis oris). (A, C) Preoperatively. (B, D) Nine months after the procedure, the patient has enduring aesthetic benefits, including eversion of the upper and lower lateral oral mucosa, shortening of the lateral upper lip, enhanced definition of the cupid’s bow, restored philtral columns, and more distinct curvature of the cutaneous surface.

**Figure 6.**
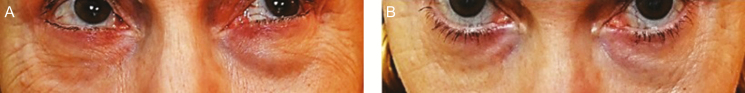
(A) This 61-year-old woman underwent Profound RF treatment of the lower lid and lateral canthus with intramuscular RF delivered into the lower and lateral orbicularis oculi (25 insertions per site). (B) Three months after Profound RF rejuvenation, cutaneous rhytids associated with contraction of the orbicularis oculi were ameliorated. Note that tightening of the orbicularis oculi manifested as reduced protrusion of the orbital septum. The patient still requires additional volume under the eyes; this could be achieved by means of filler placement, fat grafting, or orbital fat transposition.

**Figure 7.**
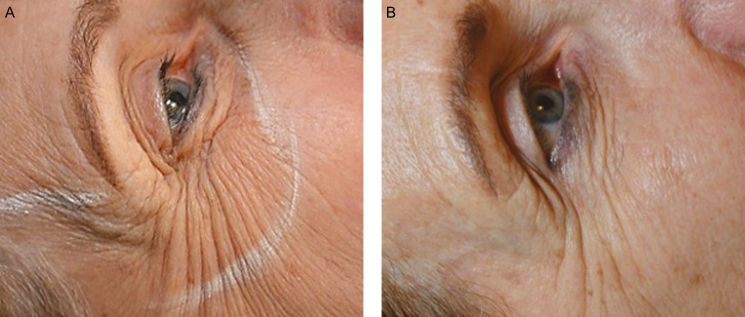
(A) This 62-year-old woman presented with facial laxity and underwent Profound RF microneedling to rejuvenate the facial skin, including the periocular, perioral, and mandibular regions as well as the forehead and neck. She also received intramuscular Profound RF treatment with 35 insertions into the lower and lateral orbicularis oculi. (B) By 6 months postoperatively, the patient appeared more youthful, with improvement in the number and severity of animation-induced rhytids surrounding the orbicularis oculi. Note the reduction in the quantity of wrinkles in the caudal–orbital and lateral–canthal regions. This outcome suggests that the orbicularis oculi has been tightened; consequently, less contraction is required to achieve ocular competency.

Facial aging is accompanied by static and dynamic skin wrinkling and laxity, which reflect volume loss and redistribution of the subcutaneous soft tissues as well as resorption and topographic changes in the underlying bony structure.^1^ The Profound RF microneedling device was scientifically designed to rejuvenate the dermis by creating fractional thermal injuries and consequently promoting the synthesis of collagen, hyaluronic acid, and elastin. When I modified the standard microneedling technique by addressing both the dermal and muscle layers of the face, I observed aesthetic benefits that surpassed those I obtained with dermal treatment alone. These included lifted and shaped brows (Figure 2 [Case 1]), an elevated oral commissure (Figure 3 [Case 2]), a shortened upper lip with vermilion eversion (Figures 1 and 3–5), restored philtral columns (Figures 3–5), reduced lower-lid fat pads (Figure 6), and fewer animation-induced wrinkles around the eye (Figure 7). Patients in my practice who undergo this modified treatment generally have decreased need for neurotoxin in the upper third of the face, with the exception of the glabella. I also have found that treatment of the upper orbicularis oris unexpectedly produces lip lift and eversion that often makes filler placement unnecessary and, for some patients, is sufficient to obviate a surgical procedure.

These findings represent early work toward addressing the mimetic musculature with bipolar RF microneedling. It should be emphasized that this modified technique has not been exhaustively optimized, and the findings described herein are preliminary. Future research is warranted to define the biochemical changes that occur in treated muscle. Collagen is a major component of the extracellular matrix of musculoskeletal tissue, but the effects of aging on musculoskeletal collagen turnover are not well understood.^14^ For instance, collagen synthesis and breakdown both appear to accelerate in old age.^14^ Further work also is needed to establish optimal procedural parameters—including temperature, time-at-temperature, and thermal lesion size^7^—for intramuscular RF application.

The primary limitation of this study is that the effect of Profound RF treatment on the musculature is demonstrated indirectly and without a validated metric. Before undertaking this investigation, I consulted 2 well-respected colleagues—a neurologist who performs facial nerve conduction studies and electromyography (EMG) and an oculoplastic surgeon—to inquire about objective assessments of mimetic muscle tightening. EMG was deemed unreliable for assessing muscle length and directional pull, and MRI was expected to have poor reproducibility for the purposes of this study. Ultimately, I concluded that it would not be possible to demonstrate the perceived alteration of muscle function with currently available modalities. Work is ongoing to identify an objective parameter that could be applied to assess change in muscle function and position.

In a pilot study, Hantash et al^7^ permitted RF microneedling treatment to proceed even when electrode placement was deeper than intended, and they subsequently obtained full-thickness biopsy specimens for histologic analysis. They found that penetration of the subcutis did not disrupt the ultrastructure of the adipose layer; instead, interstitial coagulation was noted without adipocyte necrosis.^7^ These authors did not evaluate the effects of deep insertion on cutaneous rhytids and laxity. The absence of histology in the current study precludes direct proof that the mimetic muscle is penetrated with the modified procedure. However, the length of the microneedles and the incident angle of electrode deployment as well as the presence of intraoperative bleeding and postoperative bruising support the hypothesis that the vascular musculature is being reached.

## CONCLUSIONS

Aging occurs in all layers of the face, and rejuvenation should afford a youthful appearance at rest and on animation without unnaturally altering facial expression.^5^ In a single minimally invasive microneedling session, Profound RF can be applied to the dermal and muscle layers, potentially reducing muscle redundancy and restoring collagen, elastin, and hyaluronic acid in the skin. The preliminary findings of this case study support the hypothesis that delivery of the Profound RF microneedling device into the skin and mimetic muscles yields cosmetic benefits that surpass those achieved with dermal treatment alone. Intramuscular delivery of Profound RF is a new and promising technique that warrants additional investigation.
